# Spinal low-grade ependymal tumors harboring *telomerase reverse transcriptase* promoter mutation and chromosome 7 gain with methylation profile of spinal subependymoma

**DOI:** 10.1186/s40478-026-02334-7

**Published:** 2026-06-13

**Authors:** Yoshitaka Nagashima, Junya Yamaguchi, Yusuke Nishimura, Masafumi Seki, Seiji Yamada, Sachi Maeda, Eisuke Tsukamoto, Ryo Yamamoto, Yuhei Takido, Keisuke Kimura, Fumiharu Ohka, Kennosuke Karube, Ryuta Saito

**Affiliations:** 1 Department of Neurosurgery, Nagoya University Graduate School of Medicine, 65 Tsurumai-cho, Showa-ku, Nagoya, 466-8550 Japan; 2 Department of Pathology and Laboratory Medicine, Graduate School of Medicine, Nagoya University, Nagoya, Japan; 3 Division of Analytical Pathology, Oncology Innovation Center, Research Promotion Headquarters, Fujita Health University School of Medicine, Toyoake, Japan; 4 Department of Neurological Surgery, University of California, San Francisco, CA, USA

**Keywords:** Spinal subependymoma, DNA methylation profiling, *pTERT* mutation

## Abstract

Spinal intramedullary tumors comprise a heterogeneous group of entities with diverse histopathological features, making their diagnosis particularly challenging. With the introduction of DNA methylation profiling, the underlying biological diversity of these tumors has been increasingly clarified and systematized; however, owing to the rarity of these tumors, case accumulation remains limited, and significant challenges persist. In this study, we identified two cases of spinal ependymal tumors exhibiting a methylation profile of spinal (SP-) subependymoma (SEPN). Both cases occurred in elderly patients and demonstrated circumscribed growth consistent with low-grade ependymal tumors; however, these tumors did not exhibit the typical histopathological features required for a diagnosis of SEPN in the 2021 WHO classification of central nervous system (CNS) tumors, showing indistinct cluster formation, an astrocytic immunohistochemical profile suggested by Olig2 expression, and relatively elevated Ki-67 labeling indices of 4.5% and 3.1%. At the molecular level, both cases harbored *telomerase reverse transcriptase* promoter mutations and whole chromosome 7 gain. On two-dimensional t-distributed stochastic neighbor embedding analysis, both clustered within the SP-SEPN methylation class at its periphery, with low classifier calibration scores (0.70 and 0.69). According to the current WHO classification, these cases are designated as low-grade ependymal tumors (CNS WHO grade 2) with methylation profile of SP-SEPN because they do not meet the essential WHO histopathological criteria. Ependymal tumors exhibiting a methylation profile consistent with SEPN, but discordant histopathological features have been increasingly recognized, and the appropriate classification of such tumors remains a subject of ongoing debate. These cases provide important insights into the histopathological diversity of ependymal tumors and contribute to establishing a more comprehensive and systematic classification of ependymal tumors.

**Supplementary material:**

The online version of this article (10.1186/s40478-026-02334-7) contains supplementary material, which is available to authorized users.

## Introduction

 Over the past decade, the classification of brain tumors has changed dramatically. Advances in genetic analysis techniques have led to a better understanding of the molecular biology of brain tumors, and the definition and classification of brain tumors by their molecular abnormalities and DNA methylation patterns have been accepted [1]. With regard to spinal cord tumors, primary spinal intramedullary tumors are extremely rare and account for 2–4% of all central nervous system (CNS) tumors. Due to their rarity, spinal intramedullary tumors have been subjected to fewer molecular analyses compared with intracranial tumors [2].

DNA methylation profiling represents a powerful diagnostic tool that has substantially influenced the classification of CNS tumors; nevertheless, its results should always be interpreted in the broader clinical and histopathological context of individual cases [3, 4]. With the introduction of integrated diagnosis, integrating histopathological and molecular findings, ependymal tumors have emerged as one of the complex tumor types to classify [5, 6]. However, cases of ependymal tumors in which histopathological features and DNA methylation profiles do not coincide have been reported. Specifically, some tumors exhibit histological features of ependymoma but harbor a methylation profile of subependymoma (SEPN), whereas others show histological features of ependymoma but display a methylation profile of myxopapillary ependymoma [7–10]. It has been reported that the discordance rate in ependymal tumors is higher than that observed across CNS tumors, highlighting both the diagnostic challenges arising from the histopathological diversity of ependymal tumors and the limitations of the current classification system [8]. How these cases should be designated remains a matter of debate and requires further discussion.

In the current study, we performed DNA methylation profiling of spinal intramedullary tumors from our institution and identified two cases exhibiting the methylation profile of spinal (SP-) SEPN. We subsequently conducted detailed histopathological and molecular analyses of these cases, integrated with publicly available reference methylation datasets of related spinal ependymal tumor entities. Through this investigation of cases with a methylation profile of SP-SEPN, an extremely rare ependymal tumor, our study aims to contribute to the future establishment of a systematic classification of ependymal tumors.

## Materials and methods

### Patient and clinical information

This study included two cases diagnosed as corresponding to spinal low-grade glioma at the Department of Neurosurgery, Nagoya University Hospital between 2018 and 2024. Clinical data—including patient age, sex, pre- and postoperative courses, intraoperative findings, and radiological images—were retrospectively collected from medical records. In select cases, tumor specimens were obtained intraoperatively according to the research protocol and immediately frozen at − 80 °C. Additionally, all tumor specimens were processed and stored as formalin-fixed paraffin-embedded (FFPE) samples.

### Evaluation of the histopathological findings

The FFPE samples were prepared per the standard protocol. Next, slides were mounted with 5-µm thick tissue sections and stained with hematoxylin and eosin. Immunohistochemical staining was performed using antibodies against glial fibrillary acidic protein (GFAP) (Roche, polyclonal), oligodendrocyte transcription factor 2 (Olig2) (Roche, EP112 clone), isocitrate dehydrogenase (IDH) 1 R132H (Dianova GmbH, H09 clone), epithelial membrane antigen (EMA) (Roche, E29 clone), p53 (Novocastra, DO-1 clone), alpha thalassemia/mental retardation syndrome X-linked (ATRX) (Sigma-Aldrich, 2A12 clone), Ki-67 (Dako, Mib-1 clone), and MYCN proto-oncogene (MYCN) (Cell Signaling Technology, D4B2Y clone). If any of these stains were missing at the time of initial diagnosis, additional slides were prepared. All specimens were re-evaluated independently of the previous histopathological diagnosis by two pathologists (M.S. and S.Y.).

### Extraction of genomic DNA

Genomic DNA (gDNA) was extracted from frozen tumor specimens using a QIAamp DNA extraction kit (Qiagen, Hilden, Germany) or from unstained FFPE samples using a QIAamp DNA FFPE Kit (Qiagen). The concentration of gDNA was determined using Qubit (Thermo Fisher Scientific, Waltham, MA).

### Sanger sequencing and droplet digital PCR

Sanger sequencing was performed to detect point mutations of *IDH1*, *IDH2*, B-Raf proto-oncogene, serine/threonine kinase (*BRAF*), *telomerase reverse transcriptase* promoter (*pTERT*), *H3.3 histone A* (*H3F3A*) and *H3 clustered histone 2* (*HIST1H3b*). Primer sequences for these genes are listed in Supplementary Table 1. Polymerase chain reaction was performed as follows: 35 cycles with denaturation at 98 °C for 10 s, annealing at 62 °C for 30 s, and extension at 68 °C for 30 s, followed by a final extension step at 68 °C for 5 min. For the *pTERT* mutation, the extension time was modified to 1 min. Sequence analysis was performed using ApE v2.0.55.

### DNA methylation analysis

The Illumina Infinium Human Methylation EPICv2 BeadChip array (Illumina, San Diego, CA) was utilized for a comprehensive genome-wide DNA methylation analysis. The preprocessing for the analysis of the EPICv2 array data and the computation of the beta score were performed with the Minfi package and ChAMP package using R software, version 4.4.1 [11, 12]. EPICv2 array data were harmonized to 450k array data using the mLiftOver package [13]. The beta scores were normalized using the BMIQ method in the ChAMP package. The top 5000 probes exhibiting the highest median absolute deviation on the CpG islands were selected for further analysis. The methylation profiles were analyzed with two-dimensional t-distributed stochastic neighbor embedding (t-SNE) using the Rtsne package. Reference methylation data for gliomas (GSE90496, GSE264714, and GSE243240) were obtained from the Gene Expression Omnibus database (http://www.ncbi.nlm.nih.gov/geo/) [3, 14, 15]. Molecular classification and copy number analysis based on DNA methylation data were conducted using the Heidelberg Epignostix CNS Tumor Classifier (v12.8) (https://app.epignostix.com) [3].

## Results

### Clinical presentations

**Fig. 1:**
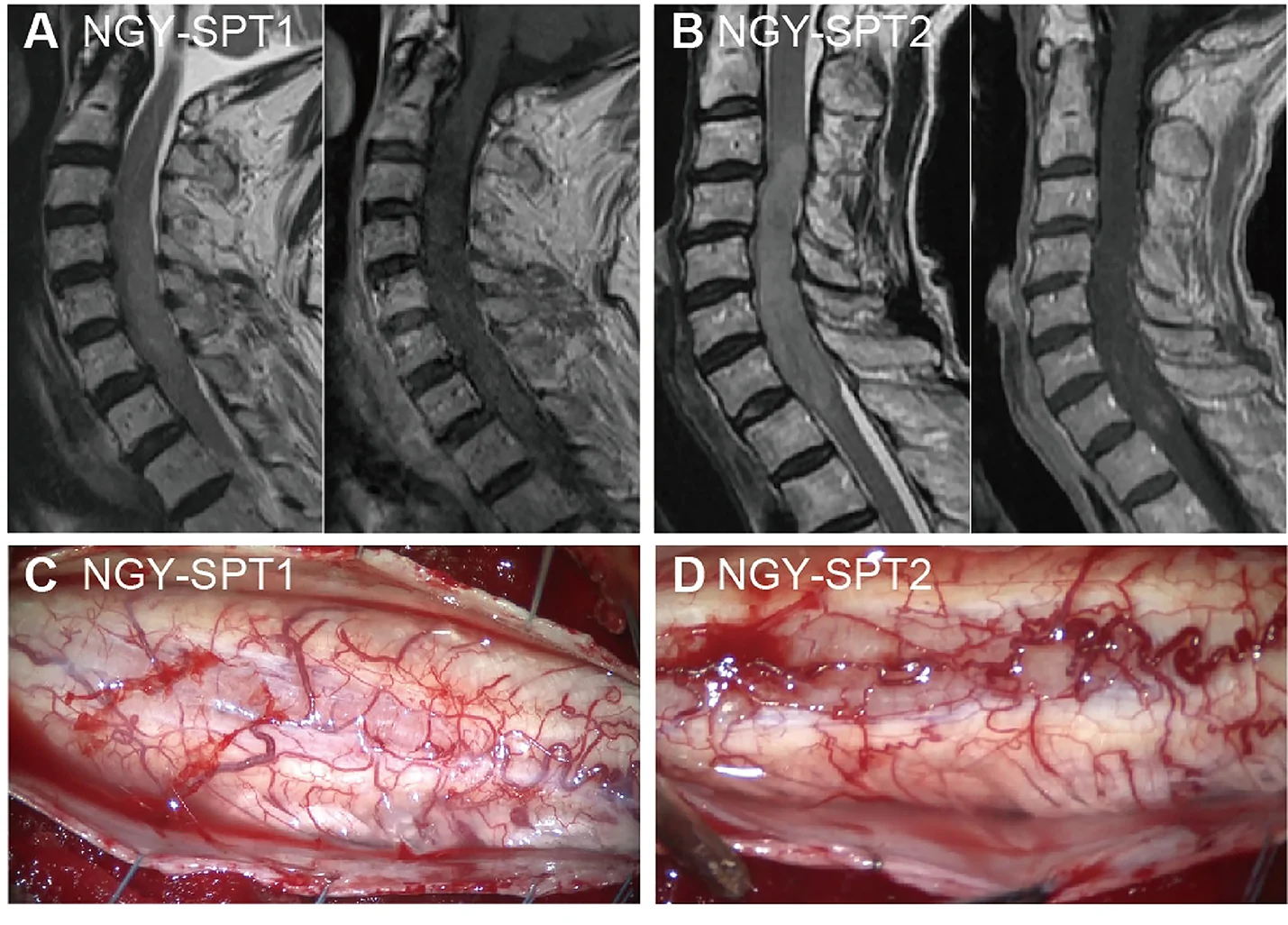
Preoperative magnetic resonance imaging (MRI) and intraoperative findings. (**A** and** B**) MRI of the two cases. T2-weighted images (left panels) show lesions that are hyperintense relative to the surrounding spinal cord parenchyma in both cases. T1 gadolinium‐enhanced images (right panels) demonstrate that the tumors are isointense to hypointense, with partial contrast enhancement observed in case NGY-SPT2. (**C** and **D**) Intraoperative findings of the spinal cord surface of two cases (NGY-SPT1 and 2). Arrows point to the part of the tumor exposed extramedullary

The patient characteristics are summarized in Table 1. At the time of surgery, patient ages were 79 and 80 years. NGY-SPT1 was male, while NGY-SPT2 was female. Both patients presented with gradually progressive neurological symptoms. Figure 1A and B demonstrated the preoperative magnetic resonance images, including T2-weighted and T1 gadolinium-enhanced images. In both cases, the lesions were located mainly in the cervical spinal cord and appeared hyperintense on T2-weighted images relative to the spinal cord parenchyma. On T1 gadolinium-enhanced images, the tumors were isointense to hypointense, with partial contrast enhancement observed in case NGY-SPT2. These radiological features were suggestive of low-grade spinal glioma. Intraoperative the spinal cord exhibited a change in coloration around the posterior median sulcus. In both cases, tumor resection was carried out, resulting in partial removal (Fig. 1C and D).

**Table 1:** Summary of patient characteristics

	Age	Gender	Location of lesion	Symptomatic period before surgery		Symptom	MRI findings of the lesions		
							T2WI	T1WI	Gd enhancement
NGY-SPT1	79	M	C3–C6	16 months		Numbness in the right upper and lower extremities	Hyper	Hypo-iso	None
NGY-SPT2	80	F	C3–Th1	60 months		Gait disturbance and bilateral hand weakness	Hyper	Hypo-iso	Partial

### Evaluation of the histopathological findings

**Fig. 2:**
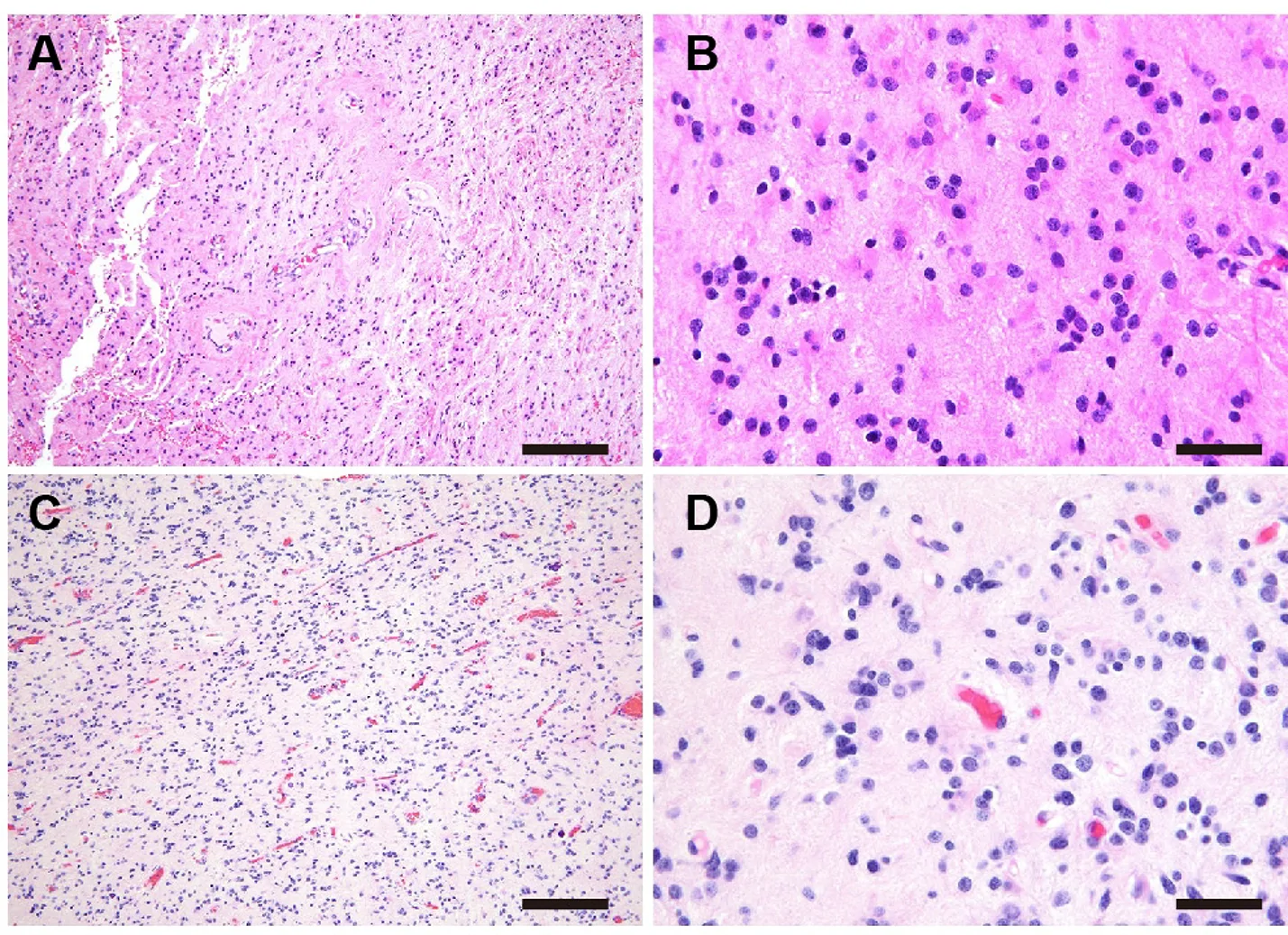
Light microscopic findings of the surgical specimens. **A**,** B** NGY-SPT1, **C**,** D** NGY-SPT2 demonstrate circumscribed growth of relatively uniform cells with round to oval nuclei. **A–D** Tumor cells form clusters amongst a rich fibrillary matrix. Original magnification: A, C x100 (scale bars = 200 μm); B, D x400 (Scale bars = 50 μm)

On histopathological examination, NGY-SPT1 and 2 presented relatively similar features: circumscribed tumors showing a proliferation of atypical glial cells with relatively uniform round to oval nuclei, forming clusters among a fibrillary matrix (NGY-SPT1, Fig. 2A and B; NGY-SPT2, Fig. 2C and D). In NGY-SPT1 and 2, eosinophilic cytoplasm was detected and was especially conspicuous in NGY-SPT1. Both tumors showed perivascular arrangement with an anucleate zone. Microcystic change was not seen. No mitotic figures or necrosis were detected.

**Fig. 3:**
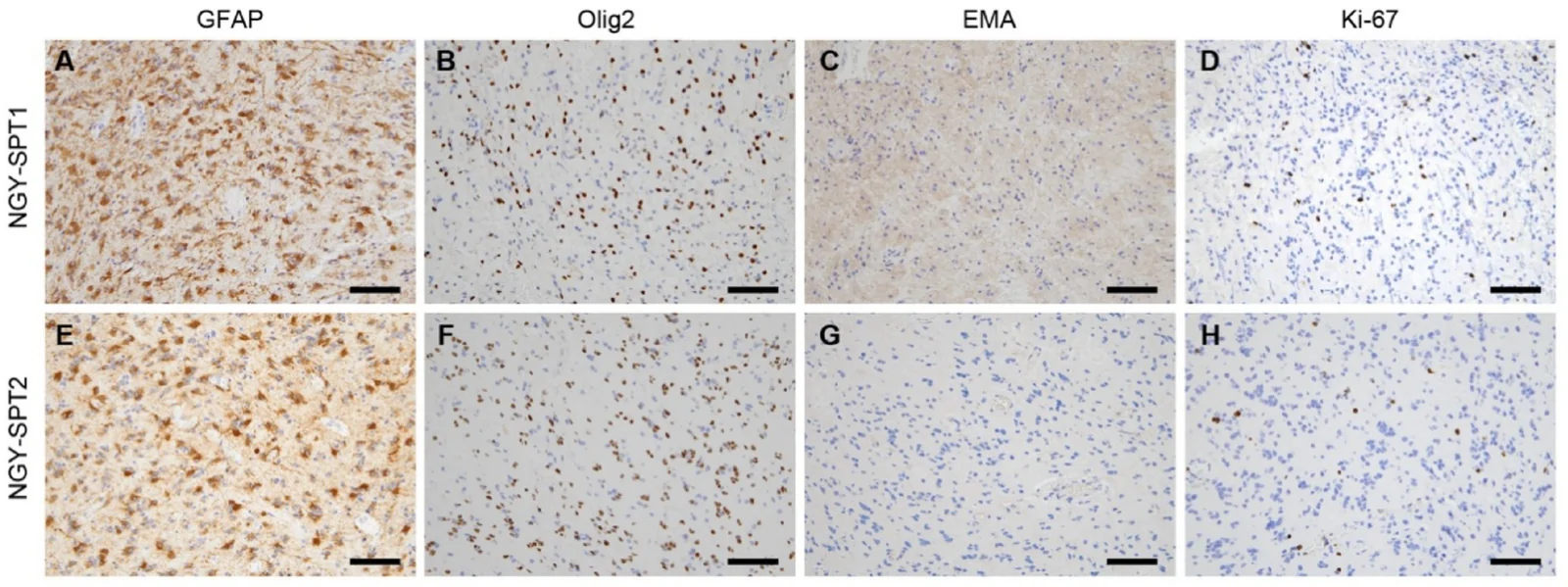
Immunohistochemical staining of the surgical specimens. GFAP (**A** and** E**), Olig2 (**B** and** F**), EMA (**C** and** G**) and Ki-67 (**D** and** H**) in the surgical specimens of NGY-SPT1 and 2. Original magnification, x200; scale bars = 100 μm

Immunohistochemically, GFAP showed diffuse positivity in both tumors (Fig. 3A and E). Olig2 was expressed in more than half of the tumor cells in both tumors (Fig. 3B and F). IDH1 p.R132H was negative for both tumors. Dot/ring-like EMA expression was not detected in NGY-SPT1 and 2 (Fig. 3C and G). p53 was wild-type in both tumors. ATRX expression was retained in both tumors. The results of Ki-67 labeling indices measured in hotspots for NGY-SPT1 and 2 were 4.5% and 3.1%, respectively (Fig. 3D and H).

Considering these findings, NGY-SPT1 and 2 were diagnosed histologically as low-grade ependymal tumor, CNS WHO grade 2, despite the relatively abundant cytoplasm and immunopositivity for Olig2 suggesting astrocytic features.

### Genetic and epigenetic analysis

**Fig. 4:**
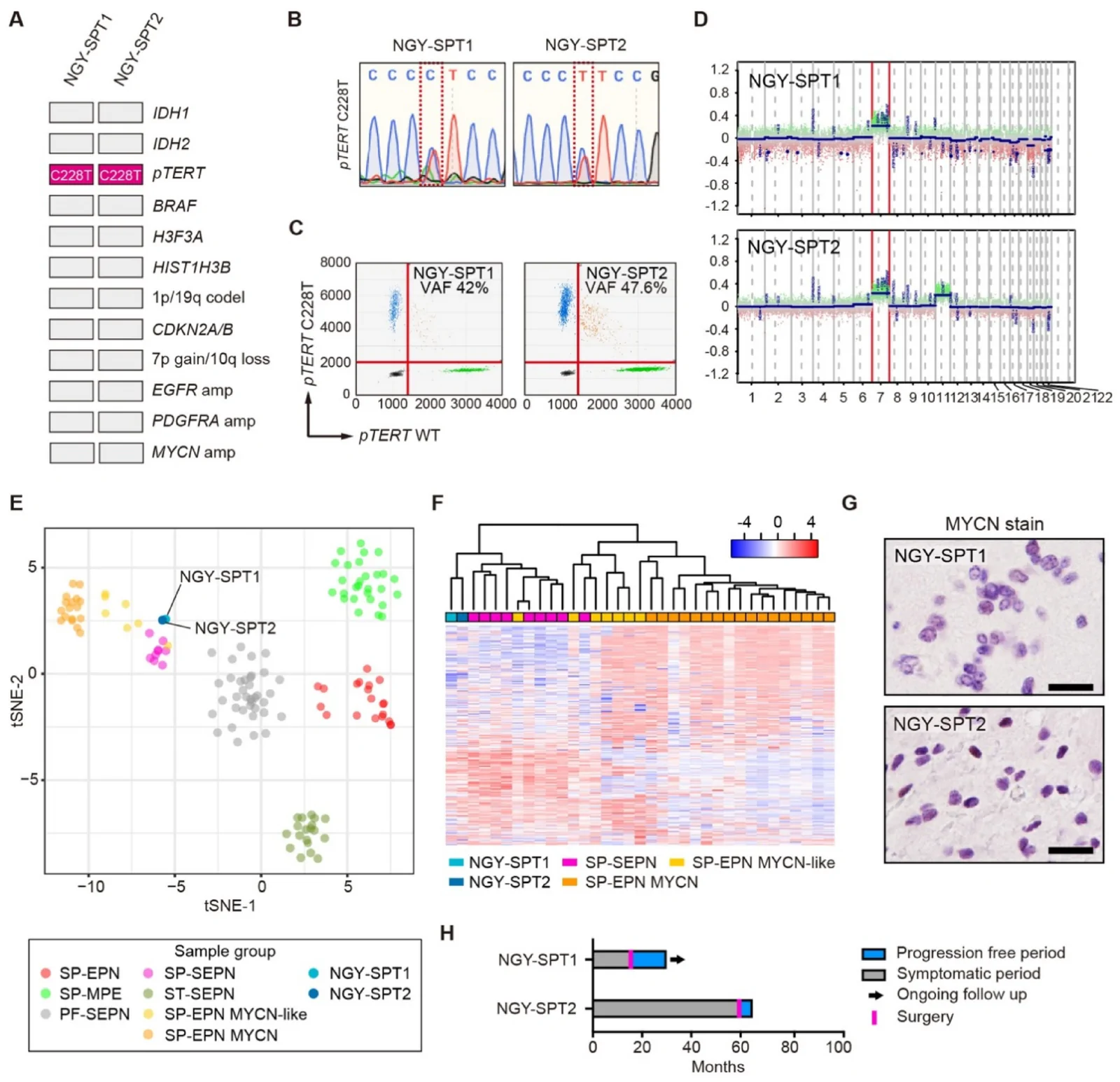
Integrated molecular profiling of the NGY-SPT1 and 2. **A** Summary of genetic findings of two cases. **B** Sanger sequencing showing *telomerase reverse transcriptase* promoter (*pTERT*) status of NGY-SPT1 and 2. Red dotted squares represent point mutations of each gene. **C** Digital droplet PCR data for *pTERT* C228T mutation (*pTERT* C228T) in NGY-SPT1 and 2. X-axis represents FAM signal of *pTERT* wild-type (WT) and Y-axis represents HEX signal of *pTERT* C228T. **D** Genome-wide copy number status analyzed from methylation array data of NGY-SPT1 and 2. Red line represents chr 7. **E** t-distributed stochastic neighbor embedding (t-SNE) plot is drawn from the methylation data of our two cases and reference data sets. Reference methylation data for central nervous system tumors (GSE90496, GSE264714, and GSE243240) were obtained from the Gene Expression Omnibus database (http://www.ncbi.nlm.nih.gov/geo/). SP-EPN, Spinal ependymoma (*n* = 20); SP-MPE, Spinal myxopapillary ependymoma (*n* = 28); PF-SEPN, Posterior fossa subependymoma (*n* = 37); SP-SEPN, Spinal Subependymoma (*n* = 9); ST-SEPN, Supratentorial Subependymoma (*n* = 19); SP-EPN MYCN-like, spinal ependymoma MYCN-like (*n* = 7); SP-EPN MYCN, spinal ependymoma MYCN (*n* = 17). **F** Hierarchical clustering based on the beta score of 5000 CpG probes in NGY-SPT1 and 2, reference data set of SP-SEPN, SP-EPN MYCN-like, and SP-EPN MYCN. **G** Immunohistochemical staining of MYCN in the surgical specimens of NGY-SPT1 and 2. Original magnification, x400; scale bars = 25 μm **H** Swimmer plot showing the clinical course of the two cases.

We performed Sanger sequencing and methylation analysis for two cases. Target Sanger sequencing assessed hotspot point mutations in six genes commonly used for diagnosis of glial tumors. The results of genetic analysis are summarized in Fig. 4A. *pTERT* mutation (C228T) was detected in NGY-SPT1 and 2 (Fig. 4B). The variant allele frequencies (VAFs) of *pTERT* C228T in NGY-SPT1 and 2 were high (42% and 47.6% respectively) (Fig. 4C). Point mutations of histone H3 and that of *BRAF* were not detected in any cases. With regard to copy number analysis, gain of whole chromosome (chr) 7 was found in NGY-SPT1 and 2, both of which harbored *pTERT* C228T mutation (Fig. 4D). 7p gain/10q loss and *EGFR* amplification as molecular features of glioblastoma, as well as *MYCN* amplification associated with the aggressive form of spinal ependymoma, were not found in any of the cases (Fig. 4A and D).

We classified these cases based on the methylation profile using the classifier [3]. NGY-SPT1 and 2 were assigned to SP-SEPN with calibration scores of 0.70 and 0.69, respectively, and were further assigned to SP-SEPN subtype A at a lower hierarchical level with calibration scores of 0.53 and 0.41 (Supplementary Table 2). NGY-SPT1 and 2 shared similar methylation profiles and clustered with SP-SEPN in the two-dimensional t-distributed stochastic neighbor embedding analysis, albeit at the periphery of the cluster of SP-SEPN (Fig. 4E). Hierarchical clustering focused on our two cases and reference set of SP-SEPN showed that NGY-SPT1 and 2 showed adjacent methylation profiles to each other but belonged to a different hierarchy than the methylation profile of the reference set of SP-SEPN (Fig. 4F). Next, we compared the methylation profiles of NGY-SPT1 and 2 with those of SP-EPN MYCN-like, which has also been reported to exhibit methylation profiles similar to SP-SEPN [14]. NGY-SPT1 and 2 exhibited methylation profiles distinct from SP-EPN MYCN-like and clustered more closely with SP-SEPN in the hierarchical clustering dendrogram (Fig. 4E and F). In addition, whereas SP-EPN MYCN-like is characterized by positive MYCN staining, NGY-SPT1 and 2 were negative for MYCN staining (Fig. 4G).

### Integrated diagnosis and prognosis

Taking the above histopathological features and molecular findings together, NGY-SPT1 and 2 were diagnosed as a low-grade ependymal tumor, CNS WHO grade 2 (with a methylation profile of SP-SEPN). For the integrated diagnosis of NGY-SPT1 and 2, a definitive diagnosis in accordance with the current WHO classification could not be established based on histopathological features and molecular findings, and given the low calibration scores, the methylation classification was reported as supplementary information, and a provisional diagnosis was assigned.

Figure 4H illustrates the clinical course before and after surgery. In patient NGY-SPT2, a gastrointestinal cancer was detected soon after surgery, and the patient died approximately five months later. The other patient experienced no significant neurological deterioration following surgery and continues to be managed in an outpatient clinic.

## Discussion

SEPN is a rare and benign CNS tumor, which occurs mainly in the lateral ventricle and fourth ventricle [16, 17]. Because SEPN exhibits distinctly different methylation profiles depending on the location of occurrence, SEPN can be divided into three subgroups depending on the location of occurrence, supratentorial (ST)-, posterior fossa (PF)- and SP-SEPN. Although the driver mutation has not been identified to date, the distinct methylation profiles suggest different biological backgrounds across these subgroups [3]. Among them, SP-SEPN is extremely rare and fewer than 150 cases have been reported to date [18]. The origin of SEPN has been debated, and progenitor glial cells differentiated from subependymal stem cells are proposed as the origin of SEPN [19, 20].

SEPN typically exhibits the clustering of small, euchromatic, and round to oval nuclei in a voluminous matrix of fibrillary cytoplasmic processes, but diverse histopathological features can be encountered, and cases exhibiting histopathological features of ependymoma (15.7–18.6% of SEPN) or astrocytoma (2.3–2.5% of SEPN) have also been recognized [21, 22]. In our cases, NGY-SPT1 and 2 did not show clear cluster formation and the presence of eosinophilic cytoplasm represented an atypical feature for SEPN. Furthermore, even relatively abundant cytoplasm and higher Olig2 expression than that observed in typical SEPN suggested astrocytic features [23]. Thus, although NGY-SPT1 and 2 harbored a methylation profile of SP-SEPN, their morphology diverged from defining SEPN features and the pathological diagnosis was confined to low-grade ependymal tumors (CNS WHO grade 2). Importantly, in NGY-SPT1 and 2 the classifier calibration scores were below the range typically considered supportive of a definitive methylation-based diagnosis, and the tumors did not meet the essential histopathological criteria for SEPN. In accordance with cIMPACT-NOW update 9 [4], we therefore interpreted the methylation result in an integrated clinicopathological context and reported the SP-SEPN methylation class assignment as supportive information rather than a basis for reclassifying these tumors as SP-SEPN.

The discordance between histopathological features and DNA methylation profiles is a known challenge, and cases with an SEPN methylation profile in which histopathological features and DNA methylation profiles do not coincide have been reported. The study of the methylation profiling of histologically defined ependymoma cases has revealed those that exhibit ependymoma-like morphology while exhibiting a methylation profile consistent with SEPN [7, 8]. In another study of posterior fossa tumors showing mixed histopathological features of SEPN and ependymoma, Thomas et al. demonstrated that such tumors that exhibit ependymoma-like morphology while exhibiting a methylation profile consistent with SEPN were shown to arise through subclonal evolution of SEPN, acquiring *pTERT* mutations and chr 6 loss, and this process was associated with more aggressive biological features [24]. Moreover, their methylation profiles shifted to patterns distinct from the original SEPN profile. Such tumors are not yet defined in the current CNS tumor classification; they have been referred to “low-grade ependymoma with methylation profile of SEPN” or “subependymoma with unusual histopathological features” or “Posterior fossa ependymal tumor with *TERT* promoter mutation and/or chromosome 6 loss and with posterior fossa subependymoma methylation class assignment, NEC” [8, 25]. SP-EPN MYCN-like has been reported to exhibit a methylation profile similar to SP-SEPN while lacking the histopathological features of SEPN. In contrast, our cases did not exhibit MYCN expression and showed a methylation profile distinct from SP-EPN MYCN-like. Furthermore, SP-EPN MYCN-like has been reported to occur in younger patients (median age in the early 30s) and to exhibit higher-grade histopathological features than those observed in our cases. Taken together, these findings indicate that our cases are distinct from SP-EPN MYCN-like. With regard to the possible tumorigenic processes underlying our cases, given that our cases exhibit methylation profiles resembling SP-SEPN, that methylation profiles are influenced by the cell of origin and acquired genetic alterations [26, 27], and that *pTERT* mutation alone are insufficient to drive tumorigenesis [28], we hypothesize that our cases may represent a tumor derived from SP-SEPN through the acquisition of secondary drivers—a *pTERT* mutation and/or chromosome 7 gain. This proposed tumorigenic model of our cases is consistent with the case reported by Thomas et al. [24]. Specifically, acquisition of a *pTERT* mutation and chromosome 7 gain may have contributed to a methylation profile distinct from typical SP-SEPN, accompanied by atypical histopathological features. This interpretation remains hypothetical and should be considered cautiously within an integrated clinicopathological framework. When the calibration score is low, it is important to consider not only the possibility that a rare genetic alteration may have caused a deviation from the established methylation profile, but also that the tumor content of the sample may be insufficient. However, in our cases, the high VAFs of the *pTERT* mutation suggested that the tumor content was adequate.

Regarding *pTERT* mutation, it is found in 7% of ependymomas and is associated with adult-onset and progression to ependymosarcoma [29]. Furthermore, this association with older age is not restricted to ependymal tumors; the *pTERT* mutation is also significantly associated with older age in other cancer types [30]. It has been reported that *pTERT* mutation in PF-SEPN is acquired in older cases and associated with the acquisition of a more aggressive phenotype [24]. In our cohort, the ages of two cases with *pTERT* mutation were 79 and 80 years, which was consistent with the association of *pTERT* mutation with older age.

The prognosis and neurological progression for low-grade ependymal tumors with *pTERT* mutation and chr 7 gain found in this study are not clear because of the small number of samples. However, the prognosis could be worse than that of tumors without these alterations. Higher Ki-67 expression in the present cases might indicate poor biology. Furthermore, a high VAF value of the *pTERT* mutation suggested that the subclone acquiring the *pTERT* mutation had outgrown and constituted the majority of the main population.

SP-SEPN is an extremely rare tumor, and it remains difficult to determine how many spinal tumors harbor the genetic alterations identified in our study while exhibiting a methylation profile of SP-SEPN. If such tumors indeed represent a subset of SP-SEPN, it is conceivable that they may have previously escaped recognition as SP-SEPN due to the absence of its characteristic histopathological features. Consequently, the true incidence of SP-SEPN may be higher than previously thought, and with the advent of molecular diagnostics, these tumors are now more likely to be accurately identified.

This study has several limitations. First, the small number of cases and the short follow-up period precluded a detailed prognostic evaluation of low-grade ependymal tumors exhibiting a methylation profile of SP-SEPN together with *pTERT* mutation and chr 7 gain. Second, the available evidence and number of cases were insufficient to establish these tumors as a distinct subset of SP-SEPN. Due to its extreme rarity and indolent biology, it is challenging for a single institution to gather a sufficient number of cases with long-term follow-up, underscoring the need for case accumulation across multiple centers.

## Conclusions

We identified two spinal low-grade ependymal tumors harboring *pTERT* mutations and chr 7 gain with a methylation profile of SP-SEPN but lacking the typical histopathological features required for a definitive diagnosis of SEPN. Although it remains a matter of debate whether these tumors should be classified as SP-SEPN, they should be recognized in the context of establishing a systematic classification of ependymal tumors.

## Supplementary material

Below is the link to the electronic supplementary material.

Supplementary file 1 (DOCX 19 kb)

Supplementary file 1 (DOCX 19 kb)

## Data Availability

Raw DNA methylation data generated in this study have been deposited in the Gene Expression Omnibus (GEO) under accession number GSE319845.

## Abbreviations

CNS: Central nervous system
SP: Spinal
SEPN: Subependymoma
FFPE: Formalin-fixed paraffin-embedded
GFAP: Glial fibrillary acidic protein
Olig2: Oligodendrocyte transcription factor 2
IDH: Isocitrate dehydrogenase
EMA: Epithelial membrane antigen
ATRX: Alpha thalassemia/mental retardation syndrome X-linked
MYCN: MYCN proto-oncogene
gDNA: Genomic DNA
BRAF: B-Raf proto-oncogene serine/threonine kinase
pTERT: Telomerase reverse transcriptase promoter
H3F3A: H3.3 histone A
HIST1H3b: Histone cluster 1
t-SNE: t-distributed stochastic neighbor embedding
VAF: Variant allele frequency
chr: Chromosome
ST: Supratentorial
PF: Posterior fossa
