# Supplementary material for: Spinal low-grade ependymal tumors harboring telomerase reverse transcriptase promoter mutation and chromosome 7 gain with methylation profile of spinal subependymoma
Source: Acta Neuropathol Commun. 2026 Jun 13;14:171. doi: 10.1186/s40478-026-02334-7 (PMC13491719; doi:10.1186/s40478-026-02334-7)
Supplement: Supplementary file 1 [file 40478_2026_2334_MOESM1_ESM.docx]

| **Supplementary table1: Primer information** | | |
| --- | --- | --- |
| Gene | F / R | Sequence |
| *IDH1* | F | 5'-CGGTCTTCAGAGAAGCCATT-3' |
|  | R | 5'-GCAAAATCACATTATTGCCAAC-3' |
| *IDH2* | F | 5'-TGTGGAAAAGTCCCAATGGA-3' |
|  | R | 5'-TGTGGCCTTGTACTGCAGAG-3' |
| *H3F3A* | F | 5'-TGCTGGTAGGTAAGTAAGGAG-3' |
|  | R | 5'-AGCAGTAGTTAAGTGTTCAAATG-3' |
| *BRAF* | F | 5'-TGCTTGCTCTGATAGGAAAATG-3' |
|  | R | 5'-AGCATCTCAGGGCCAAAAAT-3' |
| *HIST1H3B* | F | 5'-TTGGTGGTCTGACTCTATAAAAGAA-3' |
|  | R | 5'-CGGTAACGGTGAGGCTTTT-3' |
| *TERT* | F | 5'-AGTGGATTCGCGGGCACAGA-3' |
|  | R | 5'-CAGCGCTGCCTGAAACTC-3' |
