# Supplementary material for: Spinal low-grade ependymal tumors harboring telomerase reverse transcriptase promoter mutation and chromosome 7 gain with methylation profile of spinal subependymoma
Source: Acta Neuropathol Commun. 2026 Jun 13;14:171. doi: 10.1186/s40478-026-02334-7 (PMC13491719; doi:10.1186/s40478-026-02334-7)
Supplement: Supplementary file 2 [file 40478_2026_2334_MOESM2_ESM.docx]

| **Supplementary table2: Version 12.8 of the brain classifier results** | | | | | | | |
| --- | --- | --- | --- | --- | --- | --- | --- |
|  |  |  |  |  |  |  |  |
| **NGY-SPT1** | **Methylation classes** | | | | | | **Calibrated score** |
|  | Ependymal Tumours | | | | | | 0.86 |
|  |  | Spinal Subependymoma | | | | | 0.7 |
|  |  |  | Spinal Subependymoma [subtype A] | | | | 0.53 |
|  |  |  |  | Mc Subependymoma, Spinal, Subtype A | | | 0.53 |
|  |  |  | Spinal Subependymoma [subtype B] | | | | 0.16 |
|  |  |  |  | Mc Subependymoma, Spinal, Subtype B | | | 0.16 |
|  |  |  |  |  |  |  |  |
| **NGY-SPT2** | **Methylation classes** | | | | | | **Calibrated score** |
|  | Ependymal Tumours | | | | | | 0.77 |
|  |  | Spinal Subependymoma | | | | | 0.69 |
|  |  |  | Spinal Subependymoma [subtype A] | | | | 0.41 |
|  |  |  |  | Mc Subependymoma, Spinal, Subtype A | | | 0.41 |
|  |  |  | Spinal Subependymoma [subtype B] | | | | 0.27 |
|  |  |  |  | Mc Subependymoma, Spinal, Subtype B | | | 0.27 |
